# Novel Immune Subsets and Related Cytokines: Emerging Players in the Progression of Liver Fibrosis

**DOI:** 10.3389/fmed.2021.604894

**Published:** 2021-04-01

**Authors:** Minjie Wan, Jiawen Han, Lili Ding, Feng Hu, Pujun Gao

**Affiliations:** ^1^Department of Hepatology, The First Hospital of Jilin University, Jilin University, Changchun, China; ^2^Central Laboratory, The First Hospital of Jilin University, Jilin University, Changchun, China; ^3^Intensive Care Unit, The First Hospital of Jilin University, Jilin University, Changchun, China; ^4^Department of Hepatology and Gastroenterology, The Second Part of First Hospital, Jilin University, Changchun, China

**Keywords:** liver fibrosis, T helper cells, mucosa-associated invariant T cells, innate lymphoid cells, regulatory T cells, hepatic stellate cells

## Abstract

Liver fibrosis is a pathological process caused by persistent chronic injury of the liver. Kupffer cells, natural killer (NK) cells, NKT cells, and dendritic cells (DCs), which are in close contact with T and B cells, serve to bridge innate and adaptive immunity in the liver. Meanwhile, an imbalanced inflammatory response constitutes a challenge in liver disease. The dichotomous roles of novel immune cells, including T helper 17 (Th17), regulatory T cells (Tregs), mucosa-associated invariant T cells (MAIT), and innate lymphoid cells (ILCs) in liver fibrosis have gradually been revealed. These cells not only induce damage during liver fibrosis but also promote tissue repair. Hence, immune cells have unique, and often opposing, roles during the various stages of fibrosis. Due to this heterogeneity, the treatment, or reversal of fibrosis through the target of immune cells have attracted much attention. Moreover, activation of hepatic stellate cells (HSCs) constitutes the core of fibrosis. This activation is regulated by various immune mediators, including Th17, Th22, and Th9, MAIT, ILCs, and γδ T cells, as well as their related cytokines. Thus, liver fibrosis results from the complex interaction of these immune mediators, thereby complicating the ability to elucidate the mechanisms of action elicited by each cell type. Future developments in biotechnology will certainly aid in this feat to inform the design of novel therapeutic targets. Therefore, the aim of this review was to summarize the role of specific immune cells in liver fibrosis, as well as biomarkers and treatment methods related to these cells.

## Introduction

Liver fibrosis is a pathological process in which diffuse extracellular matrix (ECM) over precipitates in the liver due to abnormal hyperplasia of connective tissue caused by various pathogenic factors. The initiating event in liver fibrosis is the activation of hepatic stellate cells (HSCs), which promotes the production and accumulation of ECM (1). Liver fibrosis is a common pathological outcome of various chronic liver diseases (CLD), including chronic hepatitis B (CHB), chronic hepatitis C (CHC), non-alcoholic fatty liver disease (NAFLD), alcoholic liver disease (ALD), autoimmune hepatitis (AIH), and primary biliary cirrhosis (PBC). The treatment and prognosis of chronic liver disease depends on the degree of liver fibrosis. However, currently no treatment has demonstrated the ability to reverse the progression of fibrosis in CLD. The aggravation of fibrosis may lead to cirrhosis, liver failure, or liver cancer, in which liver transplantation is performed as the last option (2). Therefore, early detection and inhibition of fibrosis progression is particularly important in the treatment of liver diseases.

The liver is an immune organ that plays a major role in innate and adaptive immunity. Its anatomical structure allows it to function as a filter for visceral blood, thus acting as the second line of defense for the intestinal immune system, preventing the entry of harmful substances from the intestinal tract, and their negative impact throughout the body (3). The high proportion of Kupffer cells (KCs), natural killer T (NKT) cells, γδ T cells, and dendritic cells (DCs), which are in close contact with antigen presenting cells, T cells, and B cells, serve to connect innate and adaptive immunity in the liver, while inducing immune tolerance, thereby avoiding immune responses from being mounted against foreign antigens that would otherwise cause tissue damage. These effects maintain the stability of hepatic microcirculation and tolerance to foreign antigens (4). Alternatively, inflammation generally precedes fibrosis, while immune cells are important factors in the regulation of fibrosis. Although immune cells can induce damage, they can also promote tissue repair in liver fibrosis (5). T cells and macrophages constitute the core of liver fibrosis pathogenesis with macrophage-derived transforming growth factor (TGF)-β1 known to be the strongest activator of HSCs (6). Recently, newly discovered immune cells, and their related cytokines, were shown to also participate in the process of liver fibrosis (Table 1). For instance, an imbalance in the ratio of regulatory T cells (Tregs)/T helper 17 cells (Th17) is characteristic of liver fibrosis progression. Indeed, some drugs function to restore the Tregs/Th17 balance, thereby alleviated liver fibrosis (7–11). Additionally, Th22, Th9, mucosa-associated invariant T (MAIT) cells, innate lymphoid cells (ILCs), γδ T cells, and their related cytokines, have been reported to regulate liver fibrosis (12–16).

**Table 1:** The role of novel immune cells in liver fibrosis.

**Cell type**	**Species**	**Molecules and signaling pathway**	**Effect in fibrosis**	**References**
Th17	Mouse	IL-17	Pro-fibrosis	(17, 18)
	Human	IL-17	Pro-fibrosis	(20, 23, 24)
Th9	Mouse	IL-9	Pro-fibrosis	(13, 34)
MAIT	Mouse	TNF, TCR/MR1	Pro-fibrosis	(40)
	Human	IL-17A, TNF, TCR/MR1	Pro-fibrosis	(14, 40)
Treg	Mouse	CD39	Anti-fibrosis	(119)
	Human	TGF-β	Anti-fibrosis	(44)
		IL-8, TGF-β, CTLA-4	Pro-fibrosis	(45, 47)
Th22	Mouse	IL-22	Pro-fibrosis	(48, 62)
		IL-22	Anti-fibrosis	(12, 18, 61)
	Human	IL-22	Pro-fibrosis	(62, 63)
NK	Mouse	IFN-γ, RAE1/NKG20, NKp46, Ly49	Anti-fibrosis	(73, 77, 79, 80)
	Human	IFN-γ,TRAIL/NKG20, FasL/NKG20, NKp46	Anti-fibrosis	(75, 76, 79)
ILC2	Mouse	IL-33/IL-13	Pro-fibrosis	(85)
ILC3	Mouse	IL-17A, IL-22	Pro-fibrosis	(16)
γδ T	Mouse	IL-17A, CCR6/CCL20, FasL	Pro-fibrosis	(95, 96, 98)
		IFN-γ	Anti-fibrosis	(99)

*Th, Helper T cell; IL, Interleukin; MAIT, Mucosa-associated invariant T cells; TCR, T cell receptor; MR1, Major histocompatibility complex MHC class I-related molecule; TGF-β, Transforming growth factor β; NK, Natural killer cells; IFN-γ, Interferon γ; RAE, Retinoic acid early induced transcript; NKG20, natural-killer group 2, member D; TRAIL, tumor necrosis factor-related apoptosis-inducing ligand; FasL, Fas ligand; ILC, innate lymphoid cells; CCR6, CC chemokine receptor 6; CCL20, CC chemokine ligand 20*.

The aim of this review is to provide a summary of the current understanding regarding the roles of innate immune cells in liver fibrosis, and the recent diagnostic and treatment outcomes for liver fibrosis achieved through targeting newly discovered immune cells. First, the review deals with immune cells and their associated cytokines known to promote hepatic fibrosis. Second, the roles of immune cells and their related cytokines playing in anti-hepatic fibrosis are discussed. Third, the dichotomous roles of certain immune cell types in fibrosis is discussed. Finally, conclusions and future perspectives are provided.

## Immune Cells and Related Cytokines in Pro-Hepatic Fibrosis

### T Helper 17 Cells (Th17)

Th17 cells are a subset of CD4^+^ T cells characterized by RORγt expression and interleukin (IL)-17, IL-22, and IL-23 production. In acute and chronic liver injury, the amount, and proportion, of Th17 cells in the liver and peripheral blood increases. These cells have clear fibrogenic properties (17–19), with high levels of intrahepatic Th17 and IL-17 commonly observed in liver fibrosis caused by various etiologies, such as HBV (20), HCV (21), cholestatic liver injury (22), autoimmune hepatitis (23), and NAFLD (24). In fact, within a bile duct ligation (BDL) murine model, knockout of IL-17A resulted in reduced liver damage and fibrosis, accompanied by decreased levels of tumor necrosis factor (TNF)-α, TGF-β, and type I collagen in the liver compared to wild-type (WT) mice (22). Moreover, in mice with liver fibrosis induced by carbon tetrachloride (CCl4), the concentration of collagen and TGF-β in the liver of WT mice was significantly higher than in the liver of IL-17RA deficient mice. Meanwhile, *in vitro* experiments confirmed that IL-17A activates HSCs to produce collagen through the ERK1/2 and p38 signaling pathways (17). Moreover, in animal models of liver fibrosis induced by CCl4 and BDL, serum or liver IL-17 expression was positively correlated with the degree of liver fibrosis, while blocking IL-17 signaling weakened liver fibrosis. Furthermore, it has been shown that IL-17A promotes the transformation of HSCs into myofibroblasts and the production of collagen through the STAT3 signaling pathway (18).

However, Thomas et al. found that IL-17A does not directly promote HSC activation nor pro-fibrotic gene (*COL1A1, TIMP-I*, and *ACTA2*) expression, but rather requires TGF-β collaboration. Meanwhile, IL-17A upregulates and stabilizes TGF-β receptor II (TGF-βRII) expression on the surface of HSCs through the JNK signaling pathway and enhances SMAD2/3 phosphorylation to promote liver fibrosis (19). These differences may have been caused by differing experimental conditions. For instance, although both experiments conducted by Tan et al. (17) and Meng et al. (18), stimulated HSCs for 2–8 h, the latter study did not observe effects at this time point and thus, chose to further stimulate the cells for 48 h (19). Moreover, Meng et al. sought to exclude the effect of TGF-β in fetal bovine serum by performing the study under cell starvation conditions (19). Nevertheless, other studies have also confirmed that IL-17A does not induce the expression of fibrogenic genes, but rather promotes that of chemokines and pro-inflammatory factors in recruited macrophages, monocytes, and neutrophils (25, 26). Thus, IL-17 may recruit other cells to affect HSCs in complex hepatic fibrosis environments. Although advances have been made in identifying the underlying mechanism of Th17 cells and their cytokines in liver fibrosis, some challenges have arisen that require further clarification. For example, in HBV or HCV infected patients, the degree of liver fibrosis is significantly related to the virus replication rate *in vivo*. Moreover, Th17 cells and IL-17 promote viral clearance and have a certain antiviral role, similar to that of Th1 cells (27). However, both TH17 cells and IL-17 also aggravate inflammatory damage of the liver, leading to chronic HBV and HCV in patients. Due to the diverse functions of Th17 cells, determining how to exploit its anti-fibrotic effect while avoiding its pro-fibrosis potential, will serve to accelerate the clinical application of Th17 in the treatment of liver fibrosis.

### T Helper 9 Cells (Th9) and IL-9

Th9 cells are a newly distinguished CD4^+^ T cell subset characterized by the specific secretion of IL-9 and identified by PU.1 and IRF.4 (28, 29). IL-9 was originally mistaken as a type 2 cytokine until IL-4-induced differentiation of naïve CD4^+^ T cells was found to generate a group of IL-9^+^IL-10^+^Foxp3^−^ T cells with no immunosuppressive capacity (30). IL-9 was further shown to be increased in the peripheral blood and liver of mice infected with *Schistosoma japonicum*, while its inhibition reduces procollagen-III (a fibrosis-related factor) expression in infected mice (31, 32). Consistently, intraperitoneal injection of anti-IL-9 antibody inhibits granulomatous inflammation in the liver and collagen deposition around the eggs of infected mice. Furthermore, direct stimulation of HSCs *in vitro* with IL-9 significantly increases the production of collagen and α-SMA (13). In addition, Th9 cells and IL-9 are increased in the blood of patients with HBV and HBV-related cirrhosis. This elevation is also present in the liver of mice with CCl4-induced liver fibrosis (33). Furthermore, Guo et al. demonstrated that CXCL10-induced IL-9 promotes liver fibrosis via the Raf/MEK/ERK signaling pathway in CCl4-induced mice (34). Hence, Th9 has clearly been shown to promote fibrosis. Consistent with studies in liver fibrotic diseases, IL-9 antibody treatment alleviates idiopathic pulmonary fibrosis and cystic fibrosis in mice (35, 36). However, Th9 cells have only been recently identified and investigated in the context of allergic reactions and parasitic infections. Therefore, the role and mechanism of Th9 cells in liver fibrosis require further analysis.

### Mucosa-Associated Invariant T (MAIT) Cells

MAIT cells are a novel subset of innate-like T cells characterized by their invariant T cell receptor α-chain and their restrictive major histocompatibility complex related protein-1 (MR1), which are primarily distributed in the blood, liver, and intestinal mucosa (37). The innate functions of MAIT cells are similar to those of innate natural killer T cells (iNKT) and can be stimulated by IL-12 and IL-18 to secrete IFN-γ and granzyme (38). MAIT cells have antibacterial and immunological activities and present altered functions in chronic disease. The role of MAIT cells in liver fibrosis has been recognized due simply to their abundance in the liver, which accounts for ~30% of all CD3^+^ T cells present in the liver (39). In autoimmune liver disease, MAIT cells are significantly increased in the peripheral blood and liver; this increase is negatively correlated with the degree of liver fibrosis. *In vitro* studies further confirmed that IL-12 stimulates MAIT cells to produce large amounts of IL-17A. HSCs are activated by IL-17A and direct cell contact with MAIT cells, leading to HSC proliferation, pro-fibrosis, and pro-inflammatory gene expression (14). In animal models of alcoholic and non-alcoholic liver injury, MAIT cells promote the production of pro-inflammatory cytokines, such as IL-6 and IL-8 in mono-derived macrophages. Meanwhile, co-culture results demonstrate that MAIT cells promote fibroblast mitosis and pro-inflammatory properties through direct cell-cell contact (40). In addition, MR1^−/−^ mice (MAIT-deficient) are resistant to liver fibrosis and have lower fibroblast density (40). Given the abovementioned results, MAIT cells play a crucial role in the process of liver fibrosis. However, the precise associated mechanism remains to be explored.

## Immune Cells and Related Cytokines in Pro/Anti-Hepatic Fibrosis

### Regulatory T Cells (Tregs)

Tregs are a subset of immunosuppressive CD4^+^ T cells characterized by transcription factor forkhead box P3 (Foxp3) expression. The role of Tregs in liver fibrosis is complex and controversial. The number of circulating Tregs is positively correlated with the degree of liver fibrosis and serum HBV DNA load in HBV-infected patients (41). Meanwhile, Tregs inhibit HSC activation and proliferation, thereby ameliorating liver fibrosis (7, 8). However, given their immunosuppressive function, Tregs also act as a haven for hepatitis B viruses that are otherwise attacked by the immune system (41). Indeed, within HCV patients, hepatic CD4^+^Foxp3^+^ T cells are negatively correlated with liver fibrosis, whereas CD4^+^Foxp3^+^ Tregs in the blood of chronic HCV patients are less frequent than in healthy controls (42). Alternatively, Ward et al. observed no difference in the abundance of Foxp3^+^ cells between mild and severe fibrosis in portal tract areas from HCV patients (43). Moreover, it remains unclear whether hepatic Tregs directly control HSCs and immune cells in the liver, or whether Tregs in lymph nodes or the spleen suppress the activation and migration of effector cells before infiltrating into the liver. Notably, although TGF-β is a recognized pro-fibrotic factor, that produced by Tregs in HCV negatively correlates with liver inflammation and fibrosis, suggesting that TGF-β also has anti-fibrotic properties (44). The authors suggest that this dichotomy may be due to the numerous cytokines, including IL-10, that are produced by peripheral immune cells following TGF-β stimulation, thereby effectively balancing the fibrogenic effects of TGF-β produced by other cells in the liver. Additionally, IL-8^+^CD4^+^Foxp3^+^ T cells are abundant in the liver of HCV patients and are primarily distributed in the fibrosis and alpha-smooth muscle actin (α-SMA)^+^ region. Moreover, neutralization of IL-8 can block the activation of HSCs without affecting the immunosuppressive function of Tregs, suggesting that IL-8^+^ Tregs participate in the promotion of fibrosis (45). Hence different Treg subgroups appear to have opposing effects.

The mystery of Tregs in liver fibrosis is also reflected in their regulation of other immune cells. In the BDL model, Treg depletion promotes Th17 and CD8^+^ T cell infiltration in the fibrotic liver and increases the expression of inflammatory cytokines (IL-6, TNF-α, and IL-12p70) and chemokines (monocyte chemoattractant protein 1, macrophage inflammatory protein-1α, and regulated on activation, normal T-cell expressed and secreted chemokine), leading to the aggravation of fibrosis and suggesting that Tregs inhibit fibrosis by suppressing the formation of a pro-fibrotic niche by Th17 and CD8^+^ T cells (46). In contrast, Tregs are enriched in liver fibrosis tissues and protect HSCs from NK cell killing in HCV patients. Tregs inhibit NK cell killing of HSCs in two ways. The first involves inhibiting NK cells by direct contact with cytotoxic T lymphocyte associated antigen-4 (CTLA-4); whereas the second, involves the production of IL-8 and TGF-β to inhibit HSCs from producing major histocompatibility complex (MHC) class I and MHC class I chain related protein A or B (MIC-A/B), which are required for NK cell activation (47). Additionally, in severe liver fibrosis, the number of Tregs in the liver is higher than that in moderate fibrosis and is positively correlated with serum ALT levels, suggesting that Tregs may be recruited to control liver cell damage (48). In addition, the immunosuppressive regulatory effect of Tregs is conducive to the formation of chronic inflammation, which maintains liver fibrosis (49). Tregs also inhibit the secretion of matrix metalloproteinas (MMPs) by KCs through TGF-β, thereby limiting liver fibrosis regression. Meanwhile, depletion of Tregs with anti-CD25 antibodies accelerates fibrosis regression in CCl4-induced liver fibrosis mice (50). Therefore, the role of Tregs is not entirely opposed to that of Th17, and may depend on the cause of liver injury, the stage of fibrosis, and the interaction between different immune cells.

Recently, Tregs were identified in visceral adipose tissue (VAT) and are now widely accepted as associated with glucose metabolism and insulin resistance (51). In obese mice induced by a high-fat diet, metabolic syndrome and non-alcoholic steatohepatitis (NASH) occur accompanied by a decrease in the proportion of Tregs in VAT (52). VAT Tregs relieve insulin resistance and glucose metabolism disorders caused by a high-fat diet in mice (51). Conversely, consumption of VAT Tregs increases the expression of inflammatory cytokines, such as TNF-α, IL-6, and C-C chemokine ligand 5 (CCL5) and promotes insulin resistance in adipose tissues (53, 54). PPARγ, a transcription factor that regulates adipocyte differentiation, is specifically expressed in VAT Tregs (55). Disabling PPARγ on Tregs results in a decrease in VAT Tregs, while Tregs in the lymphoid organs are not affected (56). In contrast, exogenous injection of a PPAR agonist (pioglitazone) in high-fat diet mice increases the number of VAT Tregs, reduces local inflammation, and improves organic metabolism. Furthermore, mice with knocked out PPARγ expression in Tregs are less responsive to pioglitazone treatment, demonstrating that VAT Tregs constitute a key factor in the regulation of insulin sensitization (55). Considering that insulin resistance promotes the development from simple fatty liver to NASH and is a common risk factor for NAFLD (57, 58), it is reasonable to assume that VAT Tregs participate in the regulation of NASH development. Revealing the mechanism whereby VAT Tregs regulate NAFLD will, therefore, contribute to elucidating the role of VAT Tregs in liver fibrosis.

### T Helper 22 (Th22) Cells and IL-22

Th22 cells constitute a newly discovered subset of CD4^+^ effector T cells that produce a high level of IL-22 rather than IL-17 or interferon (IFN)-γ. These cells are induced by IL-6 and TNF from naïve CD4^+^ T cells. The characteristic transcription factor of Th22 cells is the aryl hydrocarbon receptor (AHR) (59). Th22 cells participate in chronic inflammation, autoimmune diseases, and cancers. Moreover, the IL-22 receptor (IL-22R) is a heterodimer composed of IL-22R1 and IL-10R2. Among them, IL-22R1 is primarily expressed on epithelial cells located in the skin and the lumen of the digestive and respiratory tracts, thereby determining the primary locations where IL-22 exerts its effects (60). However, the role of Th22 cells and IL-22 in liver fibrosis remains controversial. In CCl4-induced liver fibrosis mice, the proportion of Th22 cells in the spleen is higher than that in WT mice, and is accompanied by increased IL-22 levels in the serum and liver, suggesting that the microenvironment of liver fibrosis is conducive to the differentiation and proliferation of Th22 cells (61). Researchers who hold the view that IL-22 has a pro-fibrotic effect have found that IL-22 relies on MAPK to promote TGF-β signaling in HSCs and induce HSCs to produce more α-SMA (48). In patients with hepatitis B cirrhosis, the infiltration of IL-22 positive cells in the liver is significantly higher than that in healthy individuals and is positively correlated with the stage of liver fibrosis. Furthermore, in HBV transgenic mice, increased IL-22 aggravates chronic liver inflammation and fibrosis by secreting CCL10 and CCL20 to recruit Th17 cells (62). Consistently, IL-22 and IL-22(+) cells are significantly increased in the peripheral blood of HCV patients, while the number of IL-22(+) cells in the liver is positively correlated with the liver fibrosis score. Further, IL-22(+) cells are primarily distributed within the fibrotic area. *In vitro* experiments have confirmed that IL-22 inhibits LX-2 (HSC line) cell apoptosis, while promoting their proliferation as well as the production of α-SMA and collagen (63).

However, several experiments have also confirmed the anti-fibrotic and protective effects of IL-22 in the liver. IL-22 resists fibrosis by inducing senescence of activated HSCs through SCOS3, p53, and STAT3 (12). Additionally, *in vivo* injection of IL-22 in BDL mice reduces collagen α1 (I) and α-SMA production to alleviate liver fibrosis (18). Moreover, administration of IL-22 inhibits HSC activation, reduces the production of pro-inflammatory factors (IL-1β, IL-6, and TNF-α), and ameliorates liver fibrosis in CCl4-induced liver fibrosis (61). In the liver of HFD-fed mice, CXCL1 is overexpressed and promotes steatosis-to-NASH progression by inducing neutrophil infiltration, oxidative stress, and stress kinase activation. However, IL-22 treatment blocks hepatic oxidative stress and its associated stress kinases via induction of metallothionein. Furthermore, although it does not target immune cells, IL-22 treatment attenuates the inflammatory functions of hepatocyte-derived, mitochondrial DNA-enriched extracellular vesicles, thereby suppressing liver inflammation in NASH (64). The functional differences of IL-22 may be related to the diversity of its sources. Indeed, various immune cells in the liver, such as Th1, Th17, Th22, γδ T, and NKT cells can produce IL-22 (65). However, none of the abovementioned studies has identified the specific cellular source of IL-22, the identification of which may provide targets for clinical therapeutic strategies. In addition, IL-22 and IL-17 are both type 3 cytokines, which can be produced simultaneously in chronic inflammatory diseases (48). In the absence of IL-17, Th22 has a protective effect against NASH. However, in the presence of IL-17, IL-22 recruits Th17 to aggravate liver fibrosis (66). Moreover, the pro-inflammatory and anti-inflammatory role of IL-22 has been shown to be regulated by IL-17 in airway inflammation (67). Thus, determining the source of IL-22 and the effect of other cytokines, such as IL-17, on IL-22 will help us better understand the role of IL-22 in liver fibrosis.

### Innate Lymphoid Cells

ILCs are a group of heterogeneous lymphocytes involved in innate immunity. They do not express the antigen-specific receptors of T or B cells and are largely distributed at mucosal barrier sites where they participate in immune surveillance and regulation (68). ILCs are divided into three groups: Group 1 (ILC1 and NK cells, dependent on T-bet and producing IFN-γ), Group 2 (ILC2, dependent on GATA3 and RORα and producing type 2 cytokines, such as IL-13 and IL-5), and Group 3 (ILC3 and lymphoid tissue-inducing cells, dependent on RORγt and producing IL-17 and IL-22). The functions of ILC1, ILC2, ILC3, and NK cells correspond to Th1, Th2, Th17, and CD8^+^ cytotoxic T cells, respectively (69). ILCs are distributed differently in different organs. The NKP44^+^ ILC3 type predominates in the gut, where it acts as a mucosal barrier by producing IL-22. However, the NKP44^−^ ILC3 type predominates in the liver, and have the potential to differentiate into other ILCs. NKP44^−^ ILC3 is the only type present in fetal liver, while other ILCs can be detected with prolonged pregnancy (70).

The role of ILC1 in liver fibrosis, however, is yet to be reported. Nabekura et al. demonstrate the protective role of ILC1s in a mouse model of CCl4-mediated moderate acute liver injury. CCl4-mediated acute liver injury results in ATP and IL-12 production from DCs that activates ILC1s to produce IFN-γ. This results in upregulation of Bcl-2 and Bcl-xL by hepatocytes leading to reduced cell death and liver damage (71). Furthermore, Wang et al. found that group 1 ILCs in adipose tissues aggravate adipose fibrosis and promote the development of diabetes (72). However, since NK cells and ILC1 were not studied separately, the role of ILC1 in fibrosis could not be clearly defined.

As for NK cells in liver fibrosis, activated NK cells kill HSCs by producing IFN-γ (73–75). In addition, NK cells induce apoptosis of HSCs by direct cell contact, which involves Fas ligand (FasL), tumor necrosis factor-related apoptosis-inducing ligand (TRAIL), and natural killer group 2, member D (NKG2D) (76). Early activated HSCs produce large amounts of retinoic acid, leading to increased expression of RAE-1 (retinoic acid early inducible 1), a ligand that activates NK cell receptor NKG2D. RAE-1 and MICA synergically trigger NK cells to kill HSCs (77, 78). Additionally, Chamutal et al. reported that primary human and mouse HSCs express unknown ligands for human NKp46 and mouse NCR1 receptors, respectively, to mediate the killing of HSCs by NK cells (79). In addition to the activation-related receptors on NK cells, inhibitory receptors Ly-49 are also reportedly involved in NK cell killing of HSCs mediated by MHC I molecules (80, 81). Notably, NK cells preferentially help to eliminate senescent HSCs and contribute to the regression of liver fibrosis (78). Besides, other immune cells can also regulate the interaction between NK cells and HSCs. In immune stimulatory conditions, such as viral liver disease or Toll-like receptor stimulation, KCs and DCs promote NK cell activation (82–84). Meanwhile, Tregs can inhibit NK cell activation to protect HSCs (47). Although the molecular mechanism underlying the NK cell anti-liver fibrosis phenomena has been extensively studied, it remains unclear whether liver resident or non-resident NK cells limit fibrosis. Moreover, the details of the interaction between NK cells and HSCs muse be further revealed before NK cells can become an immune target for anti-fibrosis strategies.

It is widely accepted that ILC2 promotes liver fibrosis. ILC2 increases at the site of hepatic fibrosis and is positively correlated with the degree of hepatic fibrosis (15). In the CCl4-induced liver fibrosis model, collagen deposition is significantly reduced following ILC2 cell depletion (85). The fibrogenic effect of ILC2 is dependent on the IL-33/IL-13 signaling pathway. Meanwhile, during chronic liver injury, increased release of IL-33 leads to the accumulation and activation of ILC2 cells in the liver through ST2 receptors on the surface of ILC2. Activated ILC2 cells then produce IL-13, which in turn activates HSCs in an IL-4Rα- and STAT6-dependent manner to aggravate liver fibrosis (85). Meanwhile, liver fibrosis is alleviated in mice lacking ST2 or IL-13, however, transfusion of ILC2 restores fibrosis (85). In addition to IL-33, thymic stromal lymphopoietin (TSLP) and IL-25 are major cytokines that drive type 2 immunity. Moreover, the activation of ILCs by the above three cytokines has been reported to lead to fibrosis in the lung and skin (86–89). Consistent with the results in other organs, simultaneously blocking IL-33, IL-25, and TSLP can improve liver and lung fibrosis and reduce IL-13 production by ILC2 in mice injected with *Schistosoma mansoni* eggs. However, other studies have found that the proportion of CD4^+^IL-13^+^ T cells increases following infection with *Schistosoma mansoni* eggs, which is accompanied by decreased ILC2 activity, suggesting that adaptive immunity may gradually replace IL-33, IL-25, and TSLP-ILC2 to maintain liver fibrosis progression (86). Although the IL-33/IL13 axis clearly promotes liver fibrosis, the specific contribution of ILC2 and type 2 immune cells remains to be investigated.

The role of ILC3 in fibrosis has only recently been discovered. In HBV patients, ILC3 is increased in peripheral blood and is positively correlated with the degree of fibrosis and inflammation. Moreover, co-culturing ILC3 with LX-2 cells demonstrated that ILC3 cells activate HSCs by producing IL-17A and IL-22. Furthermore, transferring ILC3 from normal mice to CCl4-induced *Rag-1*^−/−^ mice leads to HSC activation, ECM accumulation, and aggravation of hepatic fibrosis (16). Additionally, RORγt^+^ ILCs exert a partial protective role in the hepatic immune response induced by CCl4 (90). However, the study does not distinguish between ILC3 and lymphoid tissue-inducing cells (LTi).

LTi cells are essential for peripheral lymphoid organ and tissue development (91). These cells secrete IL-17 and IL-22 in groups, both during embryo development and after birth. Activated LTi cells also produce a large number of cytokines and chemokines during induction of peripheral lymphoid organ/tissue formation, leading to lymphocyte and DC cell aggregation (92). Therefore, LTi cells have pro-inflammatory properties and are likely to participate in the inflammatory processes associated with most diseases, including liver fibrosis. However, the specific molecular mechanisms remain to be investigated.

### γδ T Cells

γδ T cells make up 3–5% of total lymphocytes and 15–25% of T cells in the liver (93). These cells represent a double-edged sword in liver fibrosis. Wang et al. demonstrate that macrophages increase the number of IL-17A-producing γδT cells through the HMGB1-TLR4-IL-23 signaling pathway, recruit neutrophils to infiltrate the liver, and aggravate liver inflammation (94). Furthermore, in mice infected with *Schistosoma japonicum*, Vγ2 γδ T cells recruit neutrophils to granuloma and the liver by producing IL-17A, thereby aggravating liver fibrosis (95). However, γδ T cells do not only interact with immune cells to affect the fibrosis process but are also regulated by HSCs. In CCl4-induced acute liver injury and early stage liver fibrosis, exosomes released by hepatocytes bind to TLR3 and activate HSCs to produce IL-17A, which promotes the production of IL-17A by hepatic γδ T cells to aggravate liver fibrosis (96). However, it has also been argued that exosomes directly promote the production of IL-17 by γδ T cells (97). In addition to promoting fibrosis, γδ T cells have also been found to ameliorate liver inflammation and fibrosis. In two chronic liver injury mouse models (CCl4 and methionine-choline-deficient diet), γδ T cells are recruited to the liver through the activation of the CCR6/CCL20 signaling pathway and directly promote HSC apoptosis in a FasL-dependent manner to limit liver fibrosis (98). Liu et al. found that γδ T cells (particularly IFN-γ-producing subsets) protect the liver from fibrosis by killing activated HSCs directly or indirectly by enhancing NK cell-mediated cytotoxicity (99). Collectively, inducing the cytotoxicity of γδ T cells against HSCs can display an anti-fibrosis role, while promoting the production of IL-17 by γδ T cells aggravates fibrosis.

## Microbiota and Liver Fibrosis

The liver is exposed to gut-derived bacterial metabolites and their products through the portal vein (100). Normally, the liver maintains a delicate balance between inflammatory and regulatory immune responses. However, when gut microbiota becomes altered, microbial stimuli affect the function of immune cells in the liver and ultimately lead to the development of liver disease (101). Liver inflammation reshapes intestinal microbiota through an unknown mechanism, leading to increased *Lactobacillus* (especially *L. johnsonii*) abundance in the gut. During the recovery stage of acute liver injury induced by concanavalin A, *Lactobacillus* was found to activate intestinal ILC3 cells to produce IL-22, which repairs the intestinal mucosal barrier and blocks further metastasis of gut microbiota to the liver. Moreover, IL-22 can induce the production of IL-10 and TGF-β by recruiting regulatory DC cells to the liver to maintain immune tolerance (102). Additionally, Hendrikx et al. report that the gut microbiota regulate ILC3 cells to reduce progression of ALD. In chronic-binge ethanol feeding mice, intestinal microbiota derived AHR ligand indole-3-acetic acid are reduced, resulting in decreased IL-22 production by ILC3s. IL-22 can also regulate the expression of intestinal REG3G, which protects mice against ethanol-induced liver disease by reducing bacterial translocation. In fact, supplementation with *Lactobacillus* to produce IL-22 effectively reduces liver damage and bacterial translocation to the liver (103). Hence, considering that systemic injections of IL-22 increase the risk of hepatocellular carcinoma in patients with CLD (104–106), altering the gut microbiota to regulate the immune cells that produce IL-22 may offer a more viable option for liver injury therapeutic interventions.

In addition to ILC3, MAIT cells are also influenced by gut microbiota in ALD. Fecal extracts from patients with ALD have reduced blood MAIT cells that are hyperactivated and exhibit defective antibacterial cytokine/cytotoxic responses (37). Moreover, in an intrahepatic cholangitis model, gut *L gasseri* are enriched and translocate to the liver, where they amplify IL-17^+^ γδ T cells to promote liver fibrosis and inflammation (107). Microbial-derived lipids are presented to γδ T cell receptors through CD1d on hepatocytes, which activates γδ T cells to express IL-17A, thereby aggravating NAFLD. Notably, this is unique to hepatic γδ T cells, and cannot be applied to circulating γδ T cells (108). Overall, these studies suggest that gut microbiota helps to shape the liver immune response. Although evidence does not directly suggest that an altered gut microbiota affects the role of novel immune cells in liver fibrosis, intestinal microbiota represents the core of the gut-liver axis that has been shown to drive many liver diseases to different stages. Therefore, it has important value and far-reaching significance for research in this field.

## Chronic Inflammation in Liver Fibrosis

Inflammation is present in all the stages of liver fibrosis, cirrhosis, and hepatocellular carcinoma. Meanwhile, persistent activation of inflammatory responses contributes to the expansion of liver fibrosis (109). Degeneration and necrosis of hepatic parenchyma cells caused by various factors leads to the release of the inflammasome, which recruits and activates inflammatory cells. Activated inflammatory cells (particularly KCs) secrete TGF-β1, TNF-α, PDGF, and other factors, which promote the transformation of HSCs, or other fibrogenic cells, into myofibroblasts. Myofibroblasts continue to secrete and deposit extracellular matrix, and ultimately form hepatic fibrosis (110). Intrinsic cells and immune cells in the liver and HSCs together establish a complex regulatory system. These novel immune cells and their related cytokines do not only directly regulate HSCs and fibrogenesis, but also indirectly affect liver fibrosis by influencing the inflammatory microenvironment. For instance, IL-17 stimulates STAT3-mediated human endothelial cell activation and production of GRO-α, GM-CSF and IL-8, which regulate neutrophil recruitment to the liver (111). IL-17 can also induce HepG2 to produce IL-6 through activation of MAPK. Consequently, IL-6 stimulates Th17 cells and forms a positive feedback loop in AIH (23). Meanwhile, IL-17 recruits other inflammatory cells and promotes the synthesis of pro-inflammatory cytokines to exacerbate the inflammatory process, which triggers and maintains the differentiation of profibrogenic cells into myofibroblasts to amplify fibrosis. In addition to IL-17, TNF signaling controls NLRP3 inflammasome activation in myeloid derived cells to initiate liver inflammation, via recruitment of neutrophils and pro-inflammatory macrophages, leading to subsequent activation of fibrogenic pathways (112). Furthermore, the inhibitory regulation of Tregs favors the formation of chronic inflammation and contributes to the persistence of liver fibrosis. Specifically, Tregs suppress NK cells, M1 KCs, and CD8^+^ T cells to maintain chronic liver inflammation and fibrosis (49). Hence, application of drugs capable of regulating the liver immune microenvironment while inducing the related cells to support the reversal of liver fibrosis may represent a new strategy for treating liver fibrosis.

## Clinical Relevance

The novel immune cells discussed in this review are important players in the pathogenesis of liver fibrosis. Hence, regulating their functions may represent a therapeutic strategy for the treatment of liver fibrosis. Currently, studies have shown that abrogating Th17/IL-17 signaling alleviates liver fibrosis. For instance, in *Schistosoma japonicum-*infected mice, a selective RhoA-Rho-associated kinase (ROCK) inhibitor (fasudil) limited liver fibrosis by inhibiting Th17 differentiation and IL-17 production, and upregulating Tregs (113). Moreover, abrogating inducible co-stimulator (ICOS) signaling reportedly inhibits Th17 cells, and their related cytokines, thereby reducing granulomatous inflammation and liver fibrosis around the eggs in a *Schistosoma japonicum* infection model (114). Mesenchymal stem cells have also been reported to restrict liver fibrosis by inhibiting Th17 cells (115, 116). In addition, miR-29a/miR-652, 1, 25(OH)2D3, as well as certain drugs, such as rapamycin and tofacitinib, have been shown to attenuate liver fibrosis by regulating Th17 cells (10, 11, 117, 118). Meanwhile, low dose IL-2 specifically expands and activates Treg cell populations thereby controlling autoimmune diseases and inflammation. Additionally, IL-2 and IL-2 immune complexes promote the expression of CD39 on hepatic Tregs, which inhibits the proliferation of CD8^+^ T cells and reduces the expression of osteopontin and TNF-α to diminish biliary fibrosis in murine sclerosing cholangitis (119). Cumulatively, these results provides a theoretical basis for the treatment of fibrosing cholangiopathies with low dose IL-2. In addition to limiting liver fibrosis by targeting the novel immune cells, as described above, they may also be applied for disease prediction. In fact, γδ T cells gene signature can predict the overall and recurrence free survival of patients with HCC. Tumor microenvironments recruit γδ T cells from peripheral or peritumor regions into tumors to elicit anti-tumor effects (120). Chronic liver inflammation and fibrosis are necessary processes in the development of HCC. Therefore, if we can effectively monitor these novel immune cells, and their related molecules, as non-invasive diagnostic markers, it will be of great benefit to patients with chronic liver disease.

## Conclusions

**Figure 1:**
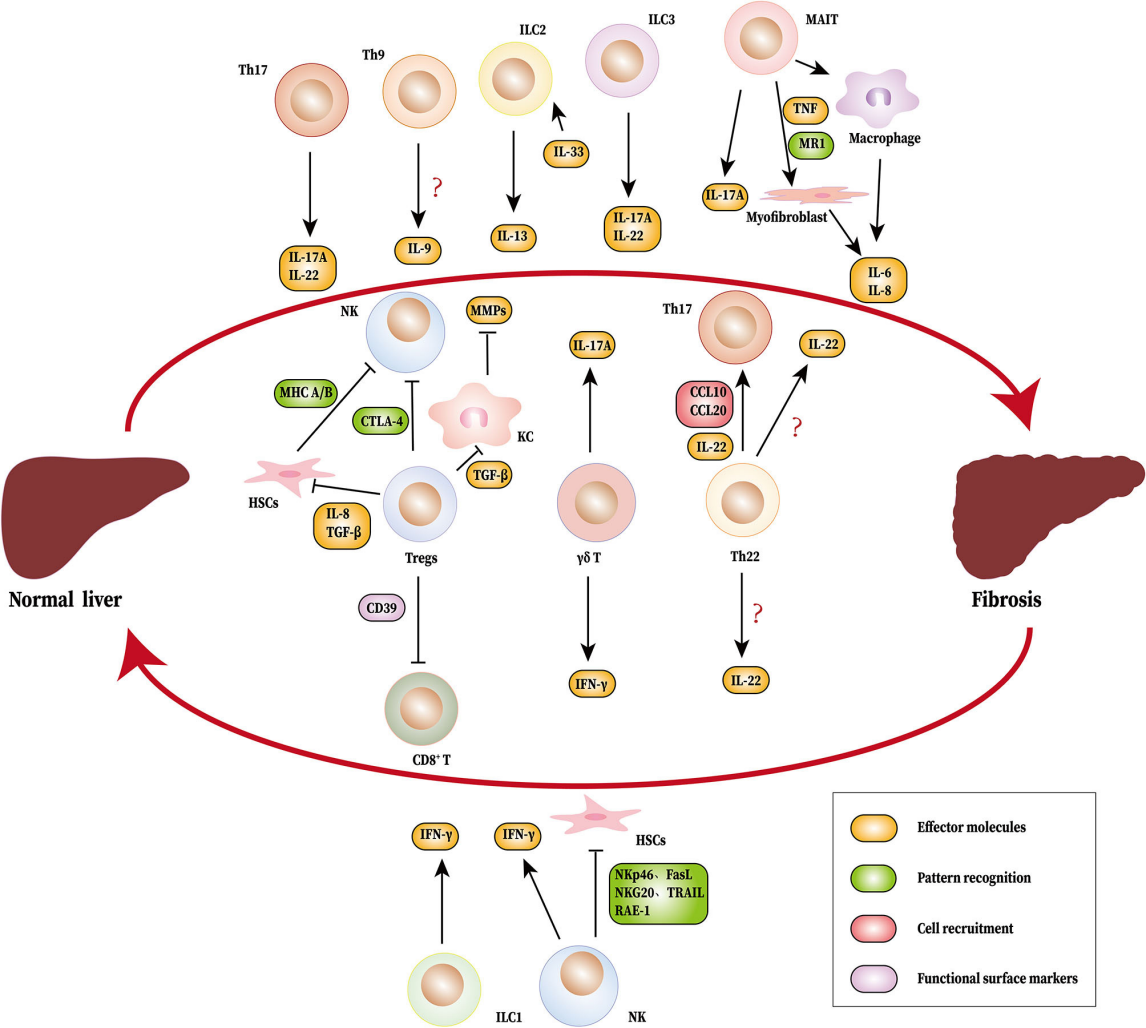
Novel immune cells and related cytokines regulate liver fibrosis. During the process of liver fibrosis, Th17 (IL-17A and IL-22), MAIT cells (IL-17A), ILC2 (IL-13), ILC3 (IL-17A, IL-22), and γδ T cells (IL-17A) promote fibrosis. MAIT cells can induce myofibroblasts and macrophages to produce IL-6 and IL-8. Tregs inhibit NK cells directly or indirectly by HSCs. Tregs also suppress KC to produce MMPs by TGF-β. Th22 cells recruits Th17 to the liver through IL-22, CCL6, and CCL20. NK cells, ILC1 and γδ T cells produce IFN-γ, which limits fibrosis. NK cells also suppress liver fibrosis by inhibiting HSCs. The source of IL-9 and IL-22 have not been identified. HSCs, hepatic stellate cells; Th, T helper cells; MAIT cells, mucosa-associated invariant T cells; ILC, innate lymphoid cell; NK cells, natural killer cells; KC, Kupffer cells; IL, interleukin; IFN-γ, Interferon γ; TGF-β, Transforming growth factor β; MMPs, matrix metalloproteinas; RAE-1, Retinoic acid early induced transcript 1; NKG20, natural killer group 2, member D; TRAIL, tumor necrosis factor-related apoptosis-inducing ligand; FasL, Fas ligand; CCL, CC chemokine ligand; TNF, tumor necrosis factor; CTLA-4, cytotoxic T lymphocyte antigen 4; MHC A/B, major histocompatibility complex A/B.

The cellular and molecular mechanisms of liver fibrosis are currently under intense investigation. Although the reversibility of liver fibrosis provides an effective early opportunity for treatment, no ideal anti-fibrotic drug is currently available for clinical practice. The activation of HSCs constitutes the core of fibrosis and is regulated by various immune mediators. Recently, novel immune cells have been discovered whose role in liver fibrosis has also been gradually recognized (Figure 1). Additionally, the proportion of regulatory B cells (Bregs) in peripheral blood has been positively correlated with the stage of liver fibrosis in HBV patients. Bregs inhibit effector T cells, however, enhance the function of Tregs to regulate immune tolerance in HBV-infected patients (121). In addition to regulating fibrosis by acting on HSCs, other cells also affect fibrosis controlled by the novel immune subsets and cytokines. For instance, in collaboration with TNF-α, IL-17 promotes HepG2 cells to produce more periostin, which induces fibroblasts to synthesize additional type I collagen and aggravate liver fibrosis (122). IL-17A induces intrahepatic biliary epithelial cells to undergo epithelial to mesenchymal transition, during which cells obtain fibroblast-related characteristics to promote fibrosis in PBC (123). Due to the complex microenvironment of liver fibrosis, the role of one single cell type cannot be discussed while ignoring others. For example, changes in Tregs and Th17 tend to occur simultaneously and are accompanied by Th1/Th2 shifts in the initial stages of liver fibrosis. In addition, each type of immune cell produces many different cytokines, which leads to the diversity of immune cell function. Fortunately, the application of single-cell RNA sequencing technology (scRNA-seq) in liver disease enables us to identify some subsets of cells that are historically difficult to isolate (124). In fact, scRNA-seq has identified a specific subset of macrophages (TREM2^+^CD9^+^MNDA^+^ scar-associated macrophages) in human fibrotic liver that is primarily distributed in scarring regions. This subset promotes the production of collagen and proliferation of HSCs (125). Thus, these technologies allow us to capture information about key cell populations and discover new therapeutic targets. The development of biotechnology will facilitate the identification of new cell populations involved in liver fibrosis as well as to elucidate the mechanisms underlying liver fibrosis.

## Author Contributions

MW wrote the manuscript. JH and LD collected related literature. FH drew the figures. PG revised the manuscript. All authors contributed to the article and approved the submitted version.

## Conflict of Interest

The authors declare that the research was conducted in the absence of any commercial or financial relationships that could be construed as a potential conflict of interest.
